# Atrial fibrillation and COVID-19: an analysis of the ambulatory database

**DOI:** 10.3389/fcvm.2024.1384826

**Published:** 2024-04-16

**Authors:** Zhanna M. Sizova, Valeria L. Zakharova, Natalya N. Shindryaeva, Natalia I. Lapidus, Mariya V. Melnik, Evgenia V. Shikh, Ludmila Y. Grebenshchikova, Alexandra V. Beloborodova, Ivan P. Polovikov

**Affiliations:** ^1^ Sechenov First Moscow Medical State University, Moscow, Russia; ^2^ City Polyclinic No 2 of the Moscow Healthcare Department, Moscow, Russia; ^3^ Tver State Medical University, Tver, Russia

**Keywords:** atrial fibrillation, coronavirus infection, cardiovascular comorbidity, thromboembolic complications, retrospective analysis

## Abstract

Atrial fibrillation (AF) is the most common heart rhythm disorder in clinical practice. It worsens the quality of life of patients, leads to an increase in the mortality rate because of its association with a high risk of thromboembolic complications. The current pandemic of a new coronavirus infection, which began in March 2020, was marked by an increase in cardiovascular diseases, including an increase in the number of patients with AF. That is why it is extremely relevant to find answers to questions about the association and mutual influence of AF and coronavirus infection to reduce the risk of vascular complications. However, most research in this area has focused on hospital patients. In this study, an electronic database of outpatients with AF, including patients with a history of COVID-19 infection was analyzed in order to assess the most significant risk factors for complications.

## Introduction

The global impact of novel severe acute respiratory system—coronavirus—2 (SARS-CoV-2) pandemic on global health systems is undeniable. The activities of various communities in the context of coronavirus infection are aimed not only at the forced search for effective ways to treat and prevent SARS-CoV-2 itself, taking into consideration the emerging new strains. An equally important task is to study the features of the course of other somatic diseases, considering the possible impact of the coronavirus infection (COVID-19).

Today, the connection between (COVID-19) and cardiovascular diseases (CVD) is becoming increasingly obvious (1, 2). A retrospective analysis of data from 1,590 patients hospitalized with COVID-19 in various hospitals in China showed the presence of concomitant diseases in 25% of patients. At the same time, 16.9% of them were diagnosed with arterial hypertension (AH), 53.7% of patients were diagnosed with other CVD (Coronary heart disease (CHD), myocardial infarction (MI), cerebrovascular disease, peripherial atherosclerosis) and 8.2% of patients with diabetes mellitus (DM) (3). According to a cohort study conducted in Italy, among 22,512 patients with COVID-19, 30% of patients were diagnosed with coronary heart disease (CHD), 24.5% of patients were diagnosed with AF, 9.6% of patients with acute cerebral circulation disorder (ACCD) in the anamnesis and 35.5% of patients diagnosed with diabetes (4). In an American study conducted on 5,700 COVID-19 patients hospitalized in 12 different hospitals in New York, hypertension was diagnosed in 56.6% of patients, coronary artery disease in 11.1% of patients and diabetes in 33.8% of patients (5). According to the Russian Hospital Register of COVID-19 Patients, among the 1130 patients included in the register, 51.6% of patients had at least one of the CVD, including hypertension, which was diagnosed in 47.3% of patients, coronary artery disease was found in 9.5%, chronic heart failure (CHF)—in 9.7%, AF—in 10.1% (6).

On the other hand, there is sufficient evidence of the impact of COVID-19 on the emergence of new CVD cases (7). To refer to the cardiac manifestations of COVID-19, Hendren NS et al. suggested to introduce a new term—acute COVID-19 cardiovascular syndrome, which includes heart rhythm disturbances, acute myocardial damage, exudation into the pericardial cavity and arterial and venous thrombotic disorders—acute coronary syndrome (ACS), ACCD, pulmonary embolism (PE), deep vein thrombosis, as well as pulmonary hypertension (8).

One of the most frequent and clinically significant heart rhythm disturbances is AF. According to the epidemiological studies, AF is diagnosed in more than 33.5 million people in the world. A high incidence of AF (from 19% to 21%) is also observed among patients diagnosed with COVID-19 (9, 10). The newly diagnosed AF in patients with COVID-19, according to various sources, is found in 3.6%–6.7% of patients (11, 12).

The purpose of this study was to analyze the electronic database of outpatients with AF to identify the most significant factors in the development of complications, including patients who have had COVID-19.

## Material and methods

In preparation for the maintenance of the register of patients with AF in the City Polyclinic No. 2 of the Department of Health of Moscow, an electronic database of patients with AF was formed. The electronic database included patients observed in the clinic with a diagnosis of AF (paroxysmal or persistent/permanent form) from January 2020. For the purposes of this study, 498 patients with AF included in the database from January 2020 to April 2022 were included in the analysis.

The database contains information on demographic, anamnestic information, concomitant diseases, risk factors, the nature of the therapy, data from various laboratory and instrumental tests, information on the transferred COVID-19 and the outcomes of AF. The information was collected based on the analysis of records from outpatient medical records of patients.

The analysis does not involve any interference in the management of patients with AF and does not set out to assess the effectiveness of treatment. We evaluated the “profile” of outpatients observed in connection with AF considering the nature of the comorbidity, the outcomes of AF and possible association with COVID-19.

The results were aggregated using Medcalc® version 19.8. For descriptive statistics of quantitative data, average values for normal distribution and root mean square deviations for a distribution other than normal are used. The Mann–Whitney *U*-criterion was used to compare the data. For analytical statistics, the Mann–Whitney criterion (when comparing quantitative indicators in two subgroups), the Pearson criterion *χ*^2^ and the exact Fisher criterion (when comparing qualitative indicators) were used. The differences were considered statistically significant at *p* < 0.05.

## Results and discussion

**Figure 1:**
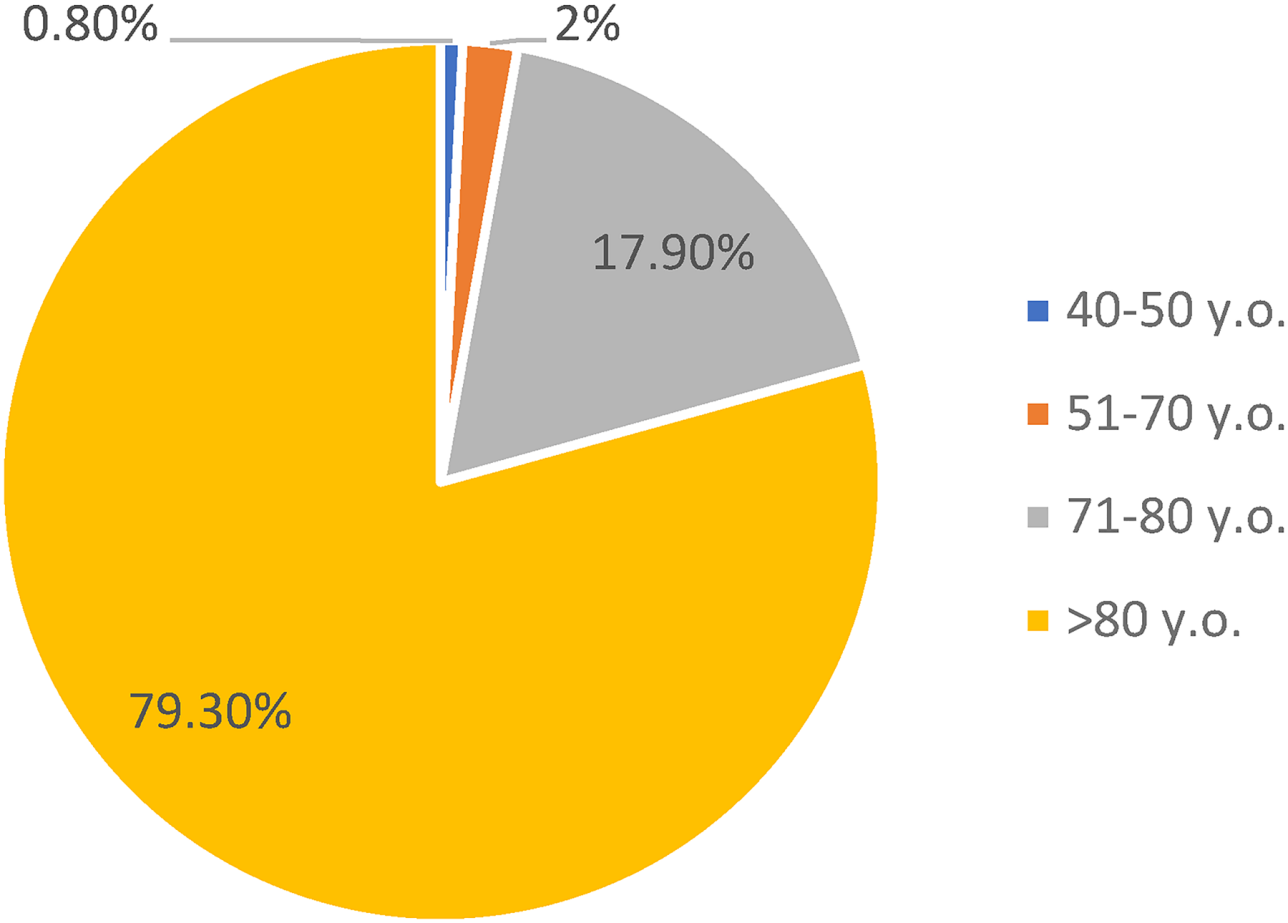
Prevalence of AF among patients of different age groups.

The database of patients with AF includes 498 patients from 41 to 97 years (average age of 82.7 ± 5.6 years). This average age of observed patients is in line with the general trends of high prevalence of AF among patients aged 65–85 (6, 13). Approximately 80% of our patients are over 80 years of age (Figure 1).

According to the gender composition among outpatients with AF, a significant predominance of women over men was revealed—357 (71.7%) vs. 141 (28.3%) accordingly, *p* < 0.05. The persistent/permanent form of AF was diagnosed in 115 patients (23.1%), paroxysmal form was diagnosed in 383 (76.9%) patients.

Table 1 provides information on other cardiovascular diseases of patients with AF included in the electronic database. Almost all patients suffering from AF have at least one more cardiovascular pathology included in the list of the most common—hypertension, coronary artery disease or CHF. In our database there was only one patient without concomitant cardiovascular pathology—a 67-year-old man with a paroxysmal form of AF without diagnosed coronary artery disease, hypertension or CHF. Thus, cardiovascular comorbidity was recorded in 497 patients with AF. Also, 34 (7.7%) patients had a history of percutaneous coronary intervention, and 13 (2.9%) patients had a history of coronary artery bypass grafting. Diabetes mellitus was detected in 60 (12%) patients included in the database.

**Table 1:** Frequency of CVD among persons with AF included in the electronic database (*n* = 498).

Cardiovascular disease	Number of patients
AH, *n* (%)	494 (99,2)
CHD, *n* (%)	441 (88,6)
Myocardial infarction (MI), *n* (%)	44 (8,8)
ACCD, *n* (%)	45 (9,0)
CHF, *n* (%)	344 (69,1)

When analyzing the risk factors for AF, smoking was detected in 25 patients (5%), obesity (body mass index > 30 kg/m^2^) in 60 patients (12%), hypodynamia in 444 patients (89.2%), hypercholesterolemia (total cholesterol > 5 mmol/L) in 481 patients (96.6%).

Patients with AF, especially the elderly, have a significantly increased risk of strokes and bleeding (14). At the same time, patients in this group are prone to increased thrombosis (15). Therefore, when prescribing drugs, it is necessary to take into consideration not only the effectiveness, but also the overall safety of these drugs. The global goals for the treatment of AF are prevention of thromboembolism, mainly strokes, and improving the quality of life by reducing symptoms and reducing the number of hospitalizations. The first goal is achieved by prescribing anticoagulants, and the second goal is achieved by controlling the rhythm or heart rate (16).

Since all patients had CHA 2 DS 2-VASc 2 or more points, they were prescribed with an anticoagulant therapy. Therefore, all patients from our database received either a vitamin K antagonist warfarin—45 (9.0%) of patients, or a thrombin inhibitor dabigatran—17 (3.4%) patients, or inhibitors of X coagulation factor apixaban or rivaroxaban—360 (72.3%) patients and 76 (15.3%) patients respectively. Control of heart rate was achieved mainly by the appointment of beta-blockers—403 (80.9%) patients received bisoprolol, nebivolol, carvedilol or metoprolol. A significant number of patients received renin-angiotensin-aldosterone system blockers (RAAS) in connection with concomitant hypertension, coronary artery disease and CHF. At the same time, angiotensin II (AII) receptor antagonists over angiotensin-converting enzyme (ACE) inhibitors significantly prevailed among the appointments—77.7% against 13.1% of appointments (*p* < 0.05). Apparently, when prescribing RAAS blockers, the attending physicians were guided mostly by their own opinion about the best safety profile and tolerability of angiotensin II (AII) receptor antagonists. Information on the nature of the therapy prescribed to patients in connection with the main and concomitant CVD is presented in Table 2.

**Table 2:** Frequency of prescribing the main groups of drugs for CVD patients with AF (*n* = 498).

Groups of drugs	Number of assignments
Any anticoagulants, *n* (%)	498 (100,0)
Incl. apixaban, *n* (%)	360 (72,3)
Rivaroxaban, *n* (%)	76 (15,3)
Dabigatran, *n* (%)	17 (3,4)
Warfarin, *n* (%)	45 (9,0)
Beta-blockers, *n* (%)	403 (80,9)
ACE inhibitors, *n* (%)	65 (13,1)
Receptor antagonists AII, *n* (%)	387 (77,7)
Calcium antagonists, *n* (%)	31 (6,2)
Statins, *n* (%)	483 (97,0)
Ezetimibe, *n* (%)	5 (1,0)
Diuretics, *n* (%)	318 (63,9)
Aldosterone antagonists, *n* (%)	300 (60,2)

Due to the fact that AF is often the cause of thromboembolic complications, we analyzed new cases of ACCD and MI since the inclusion of patients in the database.

According to the analysis, 18 (3.6%) patients developed a stroke, which for 7 of them (38.9%) was fatal. At the same time, in 5 (27.8%) patients, the stroke was repeated, 4 of which (80%) were fatal. Patients with a newly suffered stroke were slightly older than patients who, since entering into the database, had no cerebral circulation disorders—84.4 ± 4.1 vs. 82.7 ± 5.7 years (*p* > 0.05). In addition, the persistent form of AF in the group of patients with a new onset stroke was significantly more common than in the group of patients without it—11 (61.1%) patients vs. 104 (21.7%), *p* < 0.05. Persistent AF has been reported in all patients with fatal stroke. No other differences have been identified between patients in the listed above groups.

13 (2.6%) patients were diagnosed with MI during the observation. None of them had previously had a history of MI. For 3 patients (23.1%), this infarction was fatal. Significant differences between patients with a new onset MI (less than 6 months) and patients in whom MI was not registered during the observation period were not detected, with the exception of the form of AF—all patients with the new onset MI had a persistent form of AF.

Particular emphasis in our analysis was placed on patients with AF who have had a novel COVID-19. Among all the patients included in the database, 60 patients were diagnosed with COVID-19 infection, while 42 of them developed COVID-19 infection associated with a previously diagnosed AF, and 18 AF debuted against the background or after the infection.

While characterizing patients with AF who had suffered COVID-19 against the background of an already existing rhythm disturbance, it should be noted that the age of patients varied from 78 to 88 years (cf. age 83.7 ± 3.1 years), most of them were women (80.9%). And similarly, more than half of the patients (52.4%) had a permanent form of AF. A significant part of these patients had more than 2 concomitant CVD: all patients suffered from hypertension, 85.7% of patients were diagnosed with coronary artery disease (27.8% of them had a history of MI), 80.9% of patients suffered from CHF and 14.3% of patients had a history of a stroke.

Moderate and severe COVID-19, which required hospitalization due to the development of bilateral pneumonia, was diagnosed in 34 (81%) patients; 8 (19%) of which were treated on an outpatient basis. As possible causes that aggravate the severity of the course of COVID-19, we can suggest a deterioration in hemodynamics in patients with initial AF. In addition, the tendency towards hypercoagulation in COVID-19 may increase the risks of thromboembolic complications of AF. Thus, during the period of hospitalization, 8 (19%) patients died from COVID-19. 1 (2.4%) patient suffered a fatal stroke after being discharged from the hospital during the year. Thus, the total number of deaths out of all cases among patients with AF who had COVID-19 was 9 (21.4%). In addition, after suffering from COVID-19, 6 (14.3%) patients were hospitalized due to decompensation of CHF within 3–6 months after discharge from the hospital and 1 (2.4%) patient suffered from MI.

Therefore, it is obvious that both the age of patients and the accompanying cardiovascular pathology have an impact on the severity of the course of COVID-19 and its prognosis, which is consistent with the data of other researchers. Thus, the data obtained from Tai S. et al. multivariate analysis shows that the presence of concomitant CVD is an independent factor in the development of severe forms of COVID-19 (17). A viral infection can destabilize the state of the cardiovascular system, which significantly increases the risk of mortality in concomitant CVD. Atrial fibrillation is significantly associated with the risk of adverse outcomes, especially death, in patients with COVID-19 (18). According to Glybochko P. V. et al., among patients with acute respiratory distress syndrome caused by SARS-CoV-2 pneumonia, AF was associated with the development of septic shock (19).

Hypoxia and systemic inflammation that develop in patients with COVID-19 can predispose to the occurrence of AF. As noted above, in 18 of our patients, AF debuted against the background of COVID-19 (*n* = 13) or after it (*n* = 5). The average age of the patients was 84.1 ± 4.2 years, the gender composition revealed the advantage of women—12 (66.7%) vs. 6 (33.8%) patients. At the time of diagnosis of COVID-19, all 18 (100%) patients suffered from hypertension, 15 (83.3%) patients were diagnosed with coronary artery disease [3 (20%) of them had a history of MI] and 14 (77.8%) patients had CHF.

It should be noted that all these 18 patients, due to the severity of the course of COVID-19, were hospitalized. However, no lethal cases were registered. During hospitalization, 13 (72.2%) patients without initial AF had a debut of arrhythmia, and in 7 patients (53.8%), AF passed into a permanent form. In 1 patient with the paroxysmal form of AF 5 months after discharge from the hospital, ACCD was developed. In 5 (27.8%) patients without the initial AF who had COVID-19, AF was registered (2 cases—paroxysmal form, 3 cases—persistent form) within a year after recovery. In all these cases, hypertension and CHF were noted as a concomitant cardiovascular pathology in patients, and in 4 patients with coronary artery disease.

It should be noted that the occurrence of AF in patients who have a high risk of thromboembolic complications on the CHA_2_DS 2-VASc scale is an absolute indication for the appointment of anticoagulant therapy. And, according to the data in the outpatient medical records, all patients from the analyzed database received anticoagulant therapy. However, we cannot say with complete certainty that the adherence to anticoagulant treatment was fully guaranteed. It is possible that the developed thromboembolic complications were the results of disturbances in therapy. Therefore, when preparing for the maintenance of the register of patients with AF, we will consider the need to assess the adherence of patients to treatment with anticoagulants.

It is known that catheter ablation (20) and cardioversion (21) are widely used according to indications in the treatment of paroxysmal forms of atrial fibrillation. However, as we indicated in the “Materials and Methods” section, analysis of the efficacy of drug and non-drug treatment of patients was not the focus of this study.

To this date, all the pathophysiological links between COVID-19 and heart rhythm disturbances have not been sufficiently studied, but there are several assumptions. One of the possible mechanisms of this connection is implemented by changing the electrophysiological properties of atrial cells due to the development of myocardial inflammation (22). This is due to a cytokine storm and an increase in a vascular permeability (23). Another potential cause of AF is linked to an activation of sympathetic nervous system, which leads to an overload in cardiomyocytes (24). Another possible mechanism is the dysregulation of cellular ACE2 receptors caused by the COVID-19 virus. Such dysregulation leads to the release of angiotensin II, which contributes to the structural and electrical remodeling of the atrium and creates a substrate for the development of AF (25). The consequences of the described above processes are hypoxia, damage and remodeling of the myocardium, disorders of the conducting system and an increased risk of developing AF.

It is also important to mention that alongside SARS-CoV-2, there are also other viruses than may result into manifestation of AF and can be detrimental for atrial function and cause AF in a proportional way to the severity of HIV disease (26).

## Limits of the study

Due to the fact that the population selected for the study was characterized by a significant predominance of women over men—357 (71.7%) vs. 141 (28.3%) accordingly (*p* < 0.05), this may indicate a limitation in gender ratio.

As it was mentioned earlier, current database of patients with AF included 498 patients from 41 to 97 years with an average age of 82.7 ± 5.6 years. This average age of observed patients may signify comorbitity and prevalence of multiple coexisting disorders, which in turn may result into false interpretation of the results obtained by the current study and represent a bias in the study.

Because most of the patients had pre-existing CVD (arterial hypertension, CHD), this may lead to an appearance of another factor in pathogenesis of COVID-19—associated AF.

Another point of limitation is linked to a monoethnical composition of patients included in the study. Nevertheless, this also represents a significant benefit as this may show unique properties in AF clinical manifestation in the Moscow region.

## Conclusion

Atrial fibrillation is associated with adverse clinical outcomes, and above all with an increased risk of developing an ischemic stroke. The greatest importance in the development of strokes, according to the analyzed database of outpatients with AF is cardiovascular comorbidity (most often hypertension and coronary artery disease), the older age of patients, the permanent form of AF. The influence of these same factors on the severity of the course of COVID-19 and its complications was noted in patients with initial AF. Transferred COVID-19 in patients older than 75 years increases the risk of developing AF in the presence of concomitant hypertension and CHF.

## Data Availability

The original contributions presented in the study are included in the article/Supplementary Material, further inquiries can be directed to the corresponding author.
